# Neural and behavioral evidence of free shipping on consumer decision making

**DOI:** 10.1371/journal.pone.0349032

**Published:** 2026-05-13

**Authors:** Youngju Lee, Yoonsang Lee, Kyeong Seob Song, Ji-Won Chun, Dongha Lee

**Affiliations:** 1 Cognitive Science Research Group, Korea Brain Research Institute, Daegu, Republic of Korea; 2 School of Psychology, Georgia Institute of Technology, Atlanta, Georgia, United States of America; 3 Department of Medical Informatics, College of Medicine, The Catholic University of Korea, Seoul, Republic of Korea; 4 CMC Institute for Basic Medical Science, The Catholic Medical Center of The Catholic University of Korea, Seoul, Republic of Korea; Museo Storico della Fisica e Centro Studi e Ricerche Enrico Fermi, ITALY

## Abstract

The rise in online shopping has heightened consumer frustration, as many are reluctant to pay additional charges. Understanding the neural mechanisms of decision-making underlying this resistance could provide key insights into consumer behavior. However, research on these neural correlates remains limited. To address this gap, we conducted a functional magnetic resonance imaging (fMRI) study examining the relationship between shipping fee combinations and purchasing decisions. During fMRI scanning, 40 participants evaluated their intention to purchase shoes with varying shipping fees, while the total price remained constant. Behavioral measures included purchase intention rating, reaction time (RT), and the Rate Intention Score, calculated by dividing the mean purchase intention score by RT. Behavioral data revealed a clear preference for free shipping, with distinct blood-oxygenation-level-dependent (BOLD) responses in free shipping choices across shipping fee conditions. Comparisons between free shipping and the lowest shipping fee showed strong emotional preferences for free shipping, reflected in positive BOLD signals of the medial prefrontal cortex (PFC). fMRI analysis revealed that the most significant differences stemmed from variations in shipping fees, particularly between free shipping and the lowest shipping fee, highlighting the unique appeal of free shipping. As shipping fees increased, emotional influences diminished, shifting cognitive processing to the ventrolateral PFC. Additionally, increased BOLD responses in the precentral gyrus under the free-shipping option indicated its involvement in decision execution. An integrated behavioral and neural data analysis offers valuable insights into the mechanisms that influence real-world purchasing decisions.

## 1. Introduction

The rise in online shopping during the coronavirus disease 2019 pandemic enabled consumers to shop conveniently with swift doorstep delivery, avoiding physical contact or time constraints [1]. However, this convenience frequently presents significant shipping fees owing to the geographical distance between sellers and consumers. Many online retailers impose variable shipping fees, which can constitute significant portions of the total price, often reaching up to 40% [2].

Consumers may discover products with varying pricing structures, even when the overall cost remains consistent. For instance, if the product price constitutes 90% of the total, the shipping fee is 10%; conversely, if the product price constitutes 80%, the shipping cost increases to 20%. Although the total cost is identical, consumers may prefer the first option to avoid higher shipping fees and preserve perceived product value. As a strategy, online retailers itemize the total cost by presenting a lower product price separately from additional fees, rather than embedding the full cost into a single bundled product price, potentially increasing perceived cost-benefit [3–6]. In other cases, shipping or service fees are included in the total purchase cost to avoid triggering negative perceptions of surcharges [2,7]. Given the exact total cost across alternatives, do consumers prefer “free shipping” over a “cheaper product”? If so, what psychological factors drive this preference?

Several marketing studies have revealed that consumers frequently express dissatisfaction with extra charges and perceive them as unjustified or unnecessary [8–11]. This resistance to shipping fees arises from the perception of paying for something beyond the original commitment. Additionally, inconsistent structures between product prices and shipping fees can cause frustration, leading to hesitation in purchasing decisions [2,7,12]. Even when the total price remains unchanged, shipping fees continue to influence consumer choices [13].

Preferring free shipping options can also be explained by the zero-price effect. “Zero price” has been shown to exert an influence that extends beyond simply being the lowest price of an item, creating a disproportionate attraction [14,15]. Once a good’s price becomes zero, consumers tend to favor it regardless of their prior choices or preferences, which subsequently drives purchase behavior. This weighted preference for zero price—despite minimal price differences—highlights how consumers favor free options by treating them as more appealing rewards than their higher-priced counterparts [15,16]. Previous research has demonstrated strong preferences for “free” offers, particularly for free goods. Although free shipping does not imply a zero total price, assigning a zero price to one component of the price structure can still bias consumer preferences.

Research explicitly examining shipping fees by combining behavioral and neural evidence is limited. Recent consumer studies have reported that partitioning the total cost can sometimes effectively induce consumers’ purchases [9,17]. Therefore, combining neural evidence with behavioral responses highlights how consumers react to various shipping fee structures in real-life purchasing scenarios [18]. This study examines both behavioral and neural dimensions to highlight the perception of shipping fees. Investigating how neural and behavioral responses to additional charges influence consumer decision-making will provide a deeper understanding of consumers’ seemingly irrational choices.

Several neural studies on purchase decision-making processes and product preferences provide insights into the value and perception of a product [19–22]. Votinov et al. [16] reported a significant correlation between the positive evaluation of free products and activation in the reward and choice network, including the medial prefrontal cortex (mPFC) and inferior parietal lobule (IPL). Participants viewed free products more positively than priced items, thereby perceiving them as superior rewards. Similarly, Knutson et al. [23] found that excessive prices led to deactivation in the mPFC. Moreover, a product with a lower price was significantly related to activation in the lateral prefrontal cortex (PFC), which facilitated participants’ purchasing decisions [23,24]. Overall, these findings demonstrate that participants prefer free or cheaper options both emotionally and cognitively, as reflected in generalized PFC activation.

The PFC, particularly its different areas, plays a crucial role in decision-making, with specific regions activated depending on the type of decision [25]. The mPFC is involved in emotional decisions because it guides consumers to proceed with purchases once they perceive a product as valuable, regardless of whether they conduct a thorough assessment [26–28]. Conversely, the lateral PFC activity is associated with evaluating, integrating, and comparing given information [25,29,30]. The lateral PFC is involved in processing rewarding stimuli and initiating top-down control mechanisms, helping to refine decision-making by adjusting emotion-driven choices [27,31].

Given the limited neuroimaging evidence on shipping fee partitioning, this study adopted an exploratory approach to examine how behavioral preferences and neural responses vary across shipping fee structures under equal total cost. In this study, we examined how shipping fees affect online purchasing decisions using functional magnetic resonance imaging (fMRI). Specifically, we investigated whether participants’ purchasing behavior varies as a function of shipping fees. We set multiple shipping conditions based on the shipping fees that online commerce stores in the Republic of Korea typically charge. During fMRI scanning, participants completed a purchase decision task where they viewed photographs of shoes with varying price combinations and rated their purchase intentions. First, we hypothesized that a general preference for free shipping options would be observed in behavioral responses. Second, we predicted that decision-making brain regions, including the mPFC and lateral PFC, would show increased activation for free shipping options compared with paid shipping conditions. Third, we explored the relationship between behavioral responses and neural activations.

## 2. Materials and methods

### 2.1 Participants

For the experiment, we recruited 40 healthy participants through an online advertisement (22 men and 18 women, aged 20–31 years, mean = 23.3 years, standard deviation [SD] = 2.7). A sensitivity power analysis conducted using G*Power suggested that, with 40 participants, the study had a power of 80% to detect within-subject effects of Cohen’s dz ≥ 0.55 at a Bonferroni-corrected alpha level of 0.0125. All participants were right-handed, had normal or corrected-to-normal vision, and had no record of neurological or psychiatric disorders. Before scanning, participants were given detailed instructions explaining that they would evaluate their intention to purchase a pair of shoes within a fixed budget of ₩50,000. On each trial, they were asked to rate their purchase intention for shoes presented under different combinations of product price and shipping fee, with the total cost kept constant.

Before processing the MRI data, two participants were excluded owing to missing behavioral responses (> 20%). Hence, data from 38 participants (21 men and 17 women, mean age = 23.4 years, SD = 2.8) were included in the final analysis. This study was conducted in accordance with the ethical guidelines, including all methods, and approved by the Institutional Review Board of the Korea Brain Research Institute (IRB number: KBRI-202111-HR-003), and written informed consent was obtained for all participants.

### 2.2 Experimental design and procedures

**Fig 1:**
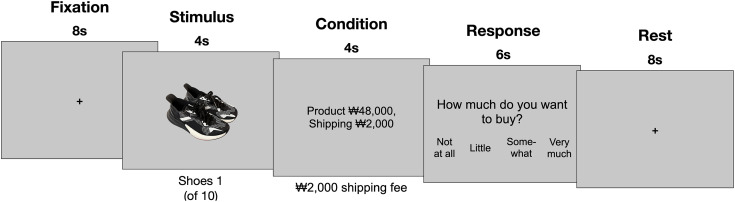
An example of a purchase decision task. Ten distinct pairs of shoes were randomly presented with five different price combinations each. A pair of shoes was presented for 4 s. Following a 4-s fixation, a price combination of shoes and shipping was presented on the screen. The pricing of each pair differs based on shipping fees ranging from ₩42,000 to ₩50,000 with ₩2,000 difference as maintaining the total price of ₩50,000 *(1. Free shipping, 2. Shipping condition 1 [*₩*2,000], 3. Shipping condition 2 [*₩*4,000], 4. Shipping condition 3 [*₩*6,000], 5. Shipping condition 4 [*₩*8,000])*. According to the price combinations, the intention to purchase was measured. Subsequently, the participants were asked to rate how much they intended to buy the shoes for the price combinations on a 4-point Likert scale (1: Not at all; 2: Little, 3: Somewhat, and 4: Very much), which was followed by 6-s rest phase.

For the fMRI experiment, participants performed a purchase decision task involving shoes (Fig 1). We prepared 10 distinct photographs of shoes and 5 different price combinations, comprising product costs and delivery charges. The shoe images were collected from publicly accessible online retail and catalog websites and were edited to ensure uniform size and resolution in a square format, with no background [32]. Any prominent logos and design elements evoking specific brands were removed [33,34]. Consequently, the shoe stimuli were designed to appear comparable in overall style and perceived quality across price conditions.

For the price combination, we first set the budget at ₩50,000 (approximately $37), which is close to the average price for a pair of shoes. Delivery fees varied between ₩0 and ₩8,000 ($6), in increments of ₩2,000 ($1.5), reflecting typical shipping charges set by most e-commerce stores in the Republic of Korea. The product prices were adjusted for each delivery fee to ensure that the total cost remained at ₩50,000 ($37). Both product cost and delivery fee were displayed in each price combination, enabling participants to focus on the overall cost. The assignment of product-delivery price combinations to shoe images was randomized across runs, such that each shoe pair was presented once under each price condition, with the order of price conditions varying across participants.

During fMRI scanning, participants completed five experimental runs, each comprising 10 trials (50 trials in total). The experiment employed a fully within-subjects design, with each participant viewing all 10 pairs of shoe images under every price combination. Each run began with an 8-s initial fixation and ended with a 2-s closing fixation. Within each trial, a pair of shoes was presented for 4 s, followed by a fixation for 4 s, a price combination for 4 s, a behavioral response period of 6 s, and an inter-trial fixation for 6 s. Each run lasted 250 s, resulting in a total task duration of approximately 21 min. Given a repetition time (TR) of 2 s, 125 functional volumes were acquired per run, yielding a total of 625 volumes across the five runs (Fig 1). For behavioral response, participants rated their intention to purchase the shoes based on the price combination by pressing one of the four buttons, each representing a level of intention on a 4-point Likert scale (1 = “Not at all” to 4 = “Very much”).

### 2.3 Data acquisition and processing

All structural and functional MRI data were acquired using a Siemens Magnetom Skyra 3.0T scanner with a 20-channel head coil (Erlangen, Germany) and a magnetization-prepared rapid gradient-echo sequence. The parameters for the high-resolution structural T1-weighted images were as follows: acquisition matrix of 256 × 256, field of view of 230 mm, voxel size of 0.7 × 0.7 × 0.7 mm³, TR of 2400 ms, and echo time (TE) of 2.3 ms. Functional imaging was performed using a T2*-weighted single-shot echo-planar (EPI) sequence with generalized auto-calibrating partially parallel acquisitions. The functional imaging parameters were as follows: acquisition matrix: 64 × 64; field of view: 192 mm; 36 interleaved slices; voxel size: 3.0 × 3.0 × 3.0 mm³; TR: 2000 ms; TE: 28 ms; flip angle: 64°; and slice gap: 0.9 mm.

All fMRI data (EPIs) were preprocessed using statistical parametric mapping (SPM12, http://www.fil.ion.ucl.ac.uk/spm/, Wellcome Trust Centre for Neuroimaging, London, UK) [35] and an in-house MATLAB toolbox (MathWorks, Inc.). All fMRI data (EPIs) underwent slice timing correction, head motion correction (realignment), co-registration of T1-weighted images to the mean EPI, and spatial normalization to convert the EPIs into the Montreal Neurological Institute template space using nonlinear transformation in SPM12. The normalized EPIs were resampled to 2.0 × 2.0 × 2.0 mm^3^ voxels, and spatial smoothing was conducted with 8 mm.

### 2.4 fMRI analysis

We focused on the differences between free and paid shipping options to examine the influence of shipping fees on purchasing decisions. We designed contrasts between the free and paid shipping options. To estimate task-evoked percent signal change in the blood-oxygen-level-dependent (BOLD) response, we performed a first-level analysis using a general linear model analysis in SPM12. Group analysis was conducted at the second level using within-subject analyses of variance (ANOVA). For statistical evaluation of the ANOVA results, we used a family-wise error (FWE) correction at the cluster level with a threshold of *p* < 0.05, following a voxel-wise threshold of *p* = 0.001.

### 2.5 Behavioral analysis

The behavioral data were analyzed based on the various price combinations. The participants’ responses, measured by their purchase intention ratings and reaction times (RTs), were analyzed using a repeated measures ANOVA in IBM SPSS Statistics for Windows, Version 24.0 (IBM Corp., Armonk, NY, USA). This analysis evaluated the main effects of purchase intention across different price combinations. The Holm–Bonferroni procedure was applied to control for multiple comparisons, with the *p*-value adjusted accordingly. Furthermore, to integrate purchase and RT, we first calculated each participant’s mean purchase intention score for each price condition. Subsequently, we divided the mean purchase intention score by the corresponding mean RT to compute the Rate Intention Score (RIS) for each participant in each price condition. In a previous study, the rate correct score (RCS) was calculated by dividing accuracy by the mean response time for each option to assess the trade-off between speed and accuracy [36]. In neuroimaging research, the RCS has been used as a behavioral measure in brain–behavior analyses [37–39]. Using a similar approach, the RIS measured the purchase intention score per unit of time.


$${RIS}_{ij}=\frac{{\text{Mean purchase intention score}}_{ij}}{{\text{Mean reaction time}}_{ij}}$$

### 2.6 Statistical analysis of behavioral scores and neural signals

To investigate the relationships between behavior and BOLD responses, we performed Pearson correlation analysis between behavior scores—including purchase intention level, RT, and the RIS—and percent signal changes in each ROI. For multiple testing of correlation *p*-values, Bonferroni correction was conducted by dividing *α* by the number of possible combinations (adjusted *p*-value = 0.001).

## 3. Results

### 3.1 Behavior analysis results

**Fig 2:**
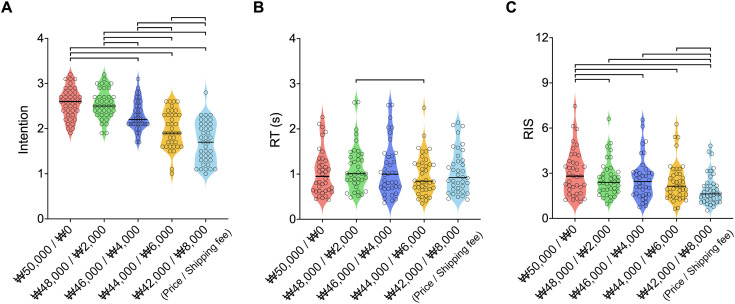
Behavioral analysis results according to the price combinations (price and shipping fee). **(A)** The purchase intention score for free shipping was higher than that for ₩4,000, ₩6,000, and ₩8,000 shipping fees, which decreased significantly as shipping fees increased. **(B)** The reaction time (RT) for the ₩6,000 shipping fee was faster than that for the ₩2,000 shipping fee. **(C)** The Rate Intention Score (RIS) shows the participants’ accurate purchase intention for each price combination by considering the purchase intention score relative to RT. Significantly higher RIS was observed for free shipping compared to the ₩2,000, ₩4,000, ₩6,000, and ₩8,000 shipping fees. Lines connecting conditions indicate statistically significant differences.

We conducted a repeated-measures ANOVA on the rating of purchase intention score and RT depending on the price combination. Significant differences were observed in the purchase intention scores depending on the price combinations, *F* (4, 148) = 63.21, η² = .63, *p* < 0.001. As shown in Fig 2A, the purchase intention score for free shipping was significantly higher than that for the ₩4,000, ₩6,000, and ₩8,000 shipping fees (*t* (37) = 4.89, *p* < 0.001; *t* (37) = 7.22, *p* < 0.001; and *t* (37) = 10, *p* < 0.001, respec*t*ively). The purchase inten*t*ion score for the ₩2,000 shipping fee was higher than that for the ₩4,000, ₩6,000, and ₩8,000 shipping fees (*t* (37) = 4.82, *p* < 0.001; *t* (37) = 6.91, *p* < 0.001; and *t* (37) = 9.51, *p* < 0.001, respectively). Addi*t*ionally, the purchase in*t*ention score for *t*he ₩4,000 shipping fee was higher than that for the ₩6,000 and ₩8,000 shipping fees (*t* (37) = 6.23, *p* < 0.001; and *t* (37) = 9.84, *p* < 0.001, respectively). Moreover, the score for the ₩6,000 shipping fee was higher than tha*t* for the ₩8,000 shipping fee (*t* (37) = 6.85, *p* < 0.001).

For the RT, significant differences were observed depending on the price combinations (*F* (4, 148) = 3.09, η^2^ = .77, *p =* 0.018). Significantly, the RT for the ₩6,000 shipping fee was faster than that for the ₩2,000 shipping fee (*t* (37) = 3.4, *p =* 0.016) (Fig 2B).

Significant differences were observed in the RIS of purchase intention depending on the price combinations (*F* (4, 148) = 18.02, η² = .33, *p* < 0.001). As shown in Fig 2C, the RIS for free shipping was significantly higher than that for the ₩2,000, ₩4,000, ₩6,000, and ₩8,000 shipping fees (*t* (37) = 3.25, *p =* 0.012; *t* (37) = 3.19, *p =* 0.0123; *t* (37) = 4.19, *p =* 0.001; and *t* (37) = 6.83, *p* < 0.001, respec*t*ively). In contrast, the RIS for the ₩8,000 shipping fee was lower than that for the ₩2,000, ₩4,000, and ₩6,000 shipping fees (*t* (37) = 5.05, *p* < 0.001; *t* (37) = 4.55, *p* < 0.001; and *t* (37) = 4.37, *p* < 0.001, respectively) (S1 Table).

### 3.2 Brain analysis results

**Fig 3:**
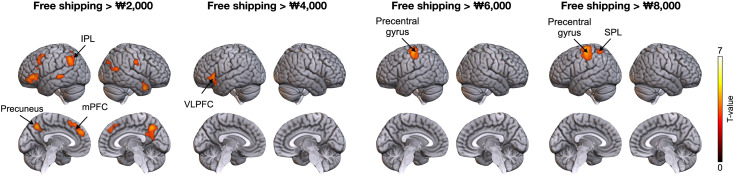
The BOLD responses related to free shipping compared with different shipping fee conditions. The inferior parietal lobule (IPL), precuneus, and medial prefrontal cortex (mPFC) showed a positive BOLD response for free shipping compared to ₩2,000 shipping. The positive activation in the ventrolateral prefrontal cortex (VLPFC) was significant in free shipping conditions compared to that in ₩4,000 shipping. The precentral gyrus showed a positive signal in free shipping conditions compared to both ₩6,000 and ₩8,000 shipping, whereas that in the superior parietal lobe was significant in free shipping compared to ₩8,000 shipping.

Fig 3 displays significant BOLD responses across cortical regions when comparing the free shipping option with each paid shipping option. Participants in the free shipping category, compared to those with a ₩2,000 shipping fee, exhibited significant positive responses in the bilateral mPFC and precuneus and left IPL (voxel-level threshold of *p* < 0.001, corrected FWE < 0.05, voxels > 251). In contrast to those with a ₩4,000 shipping fee, individuals in the free shipping category showed positive increases in the left ventrolateral prefrontal cortex (VLPFC) (voxel-level threshold of *p* < 0.001, corrected FWE < 0.05, voxels > 423). Compared to individuals with a ₩6,000 shipping fee, increased response was observed in the left precentral gyrus (voxel-level threshold of *p* < 0.001, corrected FWE < 0.05, voxels > 1267), and relative to those with a ₩8,000 shipping fee, individuals in the free shipping category exhibited increases in the left precentral gyrus and superior parietal lobule (SPL) (voxel-level threshold of *p* < 0.001, corrected FWE < 0.05, voxels > 239; Table 1).

**Table 1:** Brain regions showing statistically significant differences in free and shipping-fee conditions.

Regions (AAL abbreviation)	Peak MNI coordinates			Cluster size (mm^3^)	t−value
	x	y	z		
***Free shipping >*** ₩***2,000***					
Superior frontal gyrus L	−18	48	30	6,528	5.47
Precuneus L	−2	−58	44	8,464	5.17
Middle temporal gyrus (temporal pole) R	54	10	−30	2,000	5.05
Inferior frontal gyrus (triangular part) L	−50	38	−2	4,576	4.84
Inferior parietal gyrus L	−54	−54	44	7,280	4.78
Middle temporal gyrus L	−56	−34	0	2,240	4.48
Postcentral gyrus R	46	−12	34	2,000	4.46
Superior frontal gyrus R	16	42	36	2,248	4.27
Middle occipital gyrus R	34	−84	36	2,584	4.01
Middle frontal gyrus L	−30	20	42	2,944	4.00
***Free shipping >*** ₩***4,000***					
Inferior frontal gyrus (triangular part) L	−52	24	−2	3,384	4.53
***Free shipping >*** ₩***6,000***					
Precentral gyrus L	−36	−20	54	10,136	5.36
***Free shipping >*** ₩***8,000***					
Precentral gyrus L	−32	−20	54	15,960	6.54
Superior parietal gyrus L	−42	−54	58	1,888	3.98

AAL: Automated Anatomical Labeling atlas, L: left, R: right.

**Fig 4:**
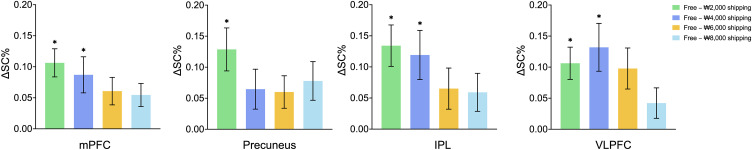
Percent signal change difference in BOLD signal between free shipping and other shipping conditions. The graphs exhibit the average percentage of signal change from the fixation baseline as a function of a stimulus. The error bars indicate the standard error of the means. Across regions of interest, free shipping elicited greater BOLD responses than other shipping conditions. Particularly, the mPFC, IPL, and VLPFC showed significantly higher signal change for free shipping than for lower paid shipping fees, indicating enhanced value-related processing when shipping costs were set to zero. * indicates *p* < 0.005.

All pairwise comparisons were evaluated using Bonferroni-corrected significance thresholds (α = 0.005, corrected for 10 comparisons). Fig 4 shows the percentage of signal change in the purchase decision-related brain regions. In the mPFC, the average percent signal change for free shipping was significantly higher than that for the ₩2,000 and ₩4,000 shipping options (free vs. ₩2,000: *t* (37) = 4.69, *p* < 0.001; free vs. ₩4,000: *t* (37) = 3.00, *p* = 0.005). The average percen*t* signal change of the precuneus was significantly higher for free shipping than for the ₩2,000 shipping option (*t* (37) = 3.72, *p* < 0.001). In *t*he IPL, the average percent signal change between free shipping and the ₩2,000 and ₩4,000 shipping options was significantly higher (free vs. ₩2,000: *t* (37) = 4.03, *p* < 0.001; free vs. ₩4,000: *t* (37) = 3.02, *p* = 0.005). Las*t*ly, the average signal change of *t*he VLPFC in free shipping was higher than that in the ₩2,000 and ₩4,000 shipping options (free vs. ₩2,000: *t* (37) = 4.10, *p* < 0.001; free vs. ₩4,000: *t* (37) = 3.43, *p* = 0.002).

### 3.3 Relationship between brain and behavior results

**Fig 5:**
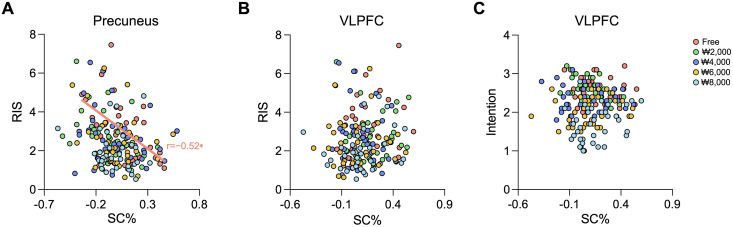
Correlation analysis results between percent signal changes (SC%) and behavioral scores (RIS and intention). The BOLD signal in the precuneus was significantly negatively correlated with the RIS in the free shipping fee condition (r = −0.52, p < 0.001). * indicates adjusted *p*-value = 0.001.

We found a significant relationship between brain and behavioral results (Fig 5). Specifically, BOLD responses in the precuneus were significantly negatively correlated with the RIS values in free shipping (*r* = −0.52, *p* < 0.001) for multiple testing (adjusted *p*-value = 0.001). Although no significance was observed in the correlation between BOLD responses and RIS values in the ₩2,000 shipping option, the precuneus showed a tendency of a negative correlation (*r* = −0.42, *p* = 0.010). The correlation of BOLD responses in the VLPFC with the RIS and purchase intention had no significance, although there was a tendency for the ₩8,000 (*r* = 0.40, *p* = 0.014) and ₩2,000 (*r* = −0.35, *p* = 0.031) shipping options.

## 4. Discussion

The evolution of online shopping has highlighted the need to assess the mechanisms underlying consumer decision-making and behavior, particularly regarding shipping fees. Our study explored behavioral and neural responses to varying shipping fees using a purchase decision task in an fMRI setting. We observed that participants consistently perceived free shipping as highly advantageous and efficient, even when the total cost was identical. However, the underlying mechanisms for choosing free shipping varied based on the shipping fee levels, indicating different cognitive processes. Free shipping appears to affect purchasing decisions in the behavior and brain, even when associated with a higher product price.

### 4.1 Behavioral pattern differences across shipping fee options

We investigated purchase intention scores and RTs to test how behavioral patterns differ according to shipping fee options. By analyzing these factors and integrating them (e.g., RIS), we found that participants significantly preferred free shipping over other shipping fee options (Fig 2C). The RIS result, which integrates purchase intention and RT, showed that free shipping consistently had the highest general preference among all shipping options in behavior. This aligns with previous findings demonstrating that consumers are more sensitive to additional charges, including shipping fees, than to base product prices [7]. Our results indicate that consumers tend to prefer free shipping, even when total prices are similar.

### 4.2 Neural pattern differences between free shipping and lower shipping fees

Participants’ considerate cognitive processes may influence decision-making, as they perceive greater benefits from the free shipping option than from the lower shipping fees. However, the underlying mechanisms of free shipping choices differed compared with those of the ₩2,000 and ₩4,000 shipping fees, as reflected in distinct neural responses. The free shipping option elicited significantly higher BOLD responses in the mPFC, IPL, and precuneus than the ₩2,000 shipping fee (Fig 3). This suggests that the free shipping choice reflects a positive and affectively driven evaluation of the option compared to a low-cost alternative [14]. These observations are consistent with those of previous studies demonstrating an association between zero-price preference and BOLD signals in the choice network comprising the mPFC and IPL [16]. Particularly, BOLD responses in the mPFC and precuneus appear to be involved in affective and preference-based decision-making, where individuals assess options based on subjective value and internal preferences [20].

However, the zero-price preference appears to operate differently as shipping fees increase. The free shipping option demonstrated significantly higher BOLD signals in the VLPFC than the ₩4,000 shipping fee (Fig 3). In this context, the preference for free shipping appears to rely more on a regulatory and deliberative decision-making process, suggesting that participants’ choices were less driven by a positive affect and more by cognitive evaluation of the price structure [40]. The VLPFC plays a crucial role in regulatory control and cognitive re-evaluation during decision-making [41–43]. Therefore, a more regulatory and considerate decision-making mechanism possibly drove participants’ preference for free shipping over the ₩4,000 shipping option.

The mPFC, precuneus, and IPL exhibited significantly higher signal changes between the free and ₩2,000 shipping options. Conversely, the VLPFC showed significantly higher signal changes between free and the ₩4,000 shipping options (Fig 4). It was expected that higher shipping fees would proportionally increase the differences in BOLD responses between free shipping and paid options. However, this pattern was not observed. Instead, we observed that valuation-related regions were more strongly engaged when free shipping was contrasted with a low fee, whereas control-related activation emerged when free shipping was contrasted with a higher fee. This pattern may be because participants perceive smaller additional fees as more of a salient nuisance, enhancing affective sensitivity to “free,” whereas higher fees prompt greater cognitive evaluation of the decision context. These findings align with those of prior research that demonstrates a strong and context-dependent preference for free options [14]. Therefore, our results indicate that while free shipping is consistently preferred, the neural processes supporting this vary depending on the comparison context.

### 4.3 Neural pattern differences between free shipping and higher shipping fees

Compared to lower shipping fees, behavior and brain responses differ when participants choose free shipping over the higher shipping fees (₩6,000 and ₩8,000). In the brain, when the free shipping option was compared to the ₩6,000 and ₩8,000 shipping fees, significantly higher BOLD responses were observed in the left precentral gyrus and SPL (Fig 3). Decision-making processes are generally related to the primary motor cortex, as individuals move through stages of comparing and evaluating options to reach a final decision [44–46]. Free shipping likely prompts participants to initiate actions consistent with their decisions, as reflected in BOLD responses of the motor area. Overall, these findings indicate that participants tend to select free shipping options when faced with high shipping fees.

### 4.4 Relationship between behavioral preference and BOLD responses

In the free and low shipping options, a higher RIS, which indicates a rapid selection of high-preference options, was associated with relatively lower BOLD responses in the precuneus (Fig 5A). The precuneus might have contributed to providing feedback for relatively favored decisions, including choosing free or low-cost shipping. The precuneus is involved in higher cognitive processes related to visual information [47,48] and the integration and comparison of values [49–51]. Our findings suggest that by engaging self-control mechanisms, increased BOLD responses in this region contribute to hesitation in purchasing decisions, even under minimal shopping costs. However, further investigation is needed to better understand the role of parietal regions in linking BOLD signals to behavioral responses during decision-making.

### 4.5 Limitations

One notable limitation is the absence of an assessment of perceptions of quality. Participants may have perceived higher-priced items as higher in quality and selected them for that reason rather than because of the shipping manipulation. Although we normalized the visual presentation of the shoes to minimize brand-related biases and kept the total price the same, it would be useful to test what people were thinking about the quality of the shoes in the images, or how that impacted the results in the brain and behavior.

The second limitation is that the RIS was introduced as an integrated behavioral index by dividing each participant’s mean purchase-intention score for each price condition by the corresponding mean RT for the same condition. Given that interpreting such a composite can be less straightforward than interpreting the component measures alone, methodological research on related integrated measures, such as the RCS, has noted that these composites can suggest effects that are unsupported by the corresponding component measures [52]. Accordingly, the RIS is interpreted as an integrated behavioral index and conclusions are drawn in conjunction with the overall pattern of the behavioral data. In addition, we didn’t assess participants’ pre- or post-preference levels for each pair of shoes. Hence, our findings may be influenced by participants’ original preferences for the products [16,41].

### 4.6 Suggestions for future research

Our study enhances the understanding of consumer response to shipping fees, a seemingly minor factor that can significantly impact decision-making. Even when the total cost remained unchanged, participants showed a consistent preference for free shipping options in their behavioral responses.

By combining behavioral and neural data, our findings demonstrate that partitioning total prices can bias consumers’ perceptions and purchasing intentions, consistent with those of prior research showing that price partitioning affects evaluation even under constant total cost conditions [12,53]. Neural findings revealed that the shipping fee structure influences the underlying reasons for varying preferences for free shipping. A small additional charge made free shipping particularly appealing. Compared to moderate shipping fees, choosing free shipping requires a considerable cognitive effort. As shipping fees increased, free shipping options tended to prompt swift decision-making.

One possible interpretation is that participants do not treat shipping fees as neutral components of total price [12,54]. This tendency may also depend on whether consumers view shipping as a seller-borne service cost or as a legitimate component of the product price. Consumers often perceive shipping fees as avoidable charges that sellers are expected to absorb as part of the service. From this perspective, free shipping functions as a salient signal of a “better deal,” enabling participants to justify their choice by avoiding an explicit loss, even when total costs are identical.

Furthermore, without needing to calculate the total price, free shipping options may become more attractive to participants. When price evaluation requires no additional computation, consumers may rely more on salient price cues, including the absence of a shipping fee. In such cases, individuals may form a strong commitment to their choice—shown here as the highest preference for free shipping—rather than engaging in a detailed evaluation of component prices [14].

Future studies can test these interpretations more directly by assessing participants’ beliefs about shipping fees. For instance, experimentally manipulating the transparency and structure of shipping fees (e.g., known vs. unknown fees and bundled vs. itemized pricing) while measuring pre- and post-choice preferences would help clarify whether free shipping preferences arise from loss avoidance, heuristic simplification, or lowered decision thresholds. Such designs would enable a more precise characterization of how representations of shipping fees shape both deliberative and automatic decision processes. They would also allow researchers to control for potential price-quality heuristics, whereby higher product prices are inferred as indicating higher quality.

This study suggests that in everyday shopping scenarios, consumers are inclined to actively seek out free shipping, frequently rationalizing the perceived value of such offers even when the difference in total cost is minimal. Understanding this cognitive dynamic is relevant for developing pricing and communication strategies in online commerce. From a seller’s perspective, bundling shipping fees into the total price would be more effective for increasing sales than presenting lower product prices accompanied by explicit surcharges. From a consumer’s standpoint, such pricing structures may reduce cognitive burden during decision-making by simplifying cost evaluation and clarifying seller responsibility for service fees, thereby facilitating easier choices. Since seemingly minor price components may disproportionately shape consumer behavior, these findings highlight the importance of considering ethical implications when designing pricing strategies. For example, offering free shipping through memberships or subscription services can boost purchases despite the total expenditure frequently exceeding the shipping cost, underscoring the need for transparency and consumer awareness alongside marketing effectiveness.

## Supporting information

S1 Table Behavioral results (*N* = 38). (DOCX)
