# Supplementary material for: Neural and behavioral evidence of free shipping on consumer decision making
Source: PLoS One. 2026 May 13;21(5):e0349032. doi: 10.1371/journal.pone.0349032 (PMC13170860; doi:10.1371/journal.pone.0349032)
Supplement: S1 Table — (DOCX) [file pone.0349032.s001.docx]

**Supplementary Table 1**. Behavioral results (*N* = 38).

| **Measure** | | **Free shipping** | **₩2,000** | **₩4,000** | **₩6,000** | **₩8,000** | ***F* score** | ***p* value** |
| --- | --- | --- | --- | --- | --- | --- | --- | --- |
| Intention | Mean | 2.57 | 2.55 | 2.28 | 1.97 | 1.69 | 63.21** | 4.5 × 10^-31^ |
|  | SE | 0.05 | 0.05 | 0.05 | 0.07 | 0.07 |  |  |
|  | 95% CI | [2.45, 2.68] | [2.45, 2.66] | [2.18, 2.39] | [1.84, 2.11] | [1.54, 1.84] |  |  |
| RT  (ms) | Mean | 1042.10 | 1143.69 | 1111.81 | 985.86 | 1052.70 | 3.09* | 0.0178 |
|  | SE | 81.26 | 83.50 | 95.97 | 73.18 | 78.69 |  |  |
|  | 95% CI | [877.46, 1206.74] | [974.51, 1312.87] | [917.36, 1306.26] | [837.58, 1134.14] | [893.26, 1212.15] |  |  |
| RIS | Mean | 3.06 | 2.65 | 2.64 | 2.45 | 1.94 | 18.02** | 4.4 × 10^-12^ |
|  | SE | 0.25 | 0.19 | 0.23 | 0.21 | 0.17 |  |  |
|  | 95% CI | [2.56, 3.56] | [2.26, 3.04] | [2.18, 3.10] | [2.02, 2.88] | [1.60, 2.28] |  |  |
| * *p* < 0.05, ** *p* < 0.001 | | | | | | | | |
